# Modern Sociology

**Published:** 1899-11-25

**Authors:** 


### Modern Sociology.

### THE EDUCATED WORKING WOMAN.

### II.—How She Might Live.

The essential requirements of the educated working woman may be set down as these: A private bedroom, a share in a public sitting-room, the use of a bathroom, good and sufficient, though plain, food, served in a clean and seemly manner; and all this at a moderate cost. Can this be obtained? Mr. Gilbert Parker says it can; and, if the experiment were carried on on a sufficiently large scale, we think it could. There is no doubt that such a house as he sketched at the Women's Congress would be immensely popular. It should be self-supporting; it must be if it is to be a success, for the flavour of charity, even of patronage, would be enough to keep the best women out of it.

For situation Westminster or Bloomsbury is suggested, the neighbourhood of Victoria Street being looked upon as the most desirable point; but in any case, the site chosen must not be beyond a twopenny 'bus fare from the chief business districts. However plain the building might be, the sanitary arrangements should be good. There should be numerous bathrooms, eight or ten on each floor, arranged side by side, and the partitions between to reach only about three-fourths of the way to the roof ceiling. One window could thus light several. The buildings should be lit by electricity, and heated by means of radiators and hot-water pipes, deriving warmth from furnaces in the basement. This would save labour, and also save residents from that awful cheerlessness that strikes the heart at going into a cold and dark room on a winter evening, and trying to light a fire with chilled fingers. There should be a dining-room, with, we hope, small tables, not the institutional long board; several drawing-rooms, a reading-room, and some small sitting-rooms. These smaller rooms might be let to such richer residents as wanted a private sitting-room, or hired, at a shilling a time, to such as desired occasionally to see friends in private, whom yet they did not wish to introduce to the intimacy of their one bed-sitting-room. But, of course, the main feature of the place must be the bedroom, the one place that the majority of the residents could call their own. The scheme comprises 400 rooms in all—280 small ones, 70 of a larger size, and 50 double bedrooms, which sisters or chums might take together. In each room there should be a recess for a hanging cupboard —it might be better if this were a regular closet, with a door after the American style, for dresses have a habit of bulging out of the best regulated curtains; and there should be abundance of shelving. The æsthetic occupant who did not want shelves for her band-boxes could fill them with china. The furniture provided should be a small sofa-bed, a chest of drawers with a looking-glass on top, a washstand, and a couple of chairs. Occupants could, of course, add rugs, draperies, and easy chairs, as their taste or means permitted. The decorations of the rooms might easily be tasteful, and yet not costly. Fortunately, good taste in wall paper and paint is not an expensive luxury. It is of importance that there should be a box-room, where boxes not in use could be kept at a small fee. This would obviate many of the inconveniences of living in one room. It is suggested that there should also be a bicycle-house where machines could be stored, and where there would be a regular cleaner, whose services could be secured at a moderate charge; but this is a detail which can hardly be settled in advance. To keep the house entirely for those for whom it is intended, it is advised that no one living entirely on private means should be allowed to live there.

As to the rates at which all this could be provided, Mr. Parker thinks the following would be adequate: For the large bedrooms, a rent of 8s. per week; for the large single ones, 7s. 6d.; and for the small single bedrooms, which are by far the majority, 5s. per week. These charges should include the free use of the dining and drawing rooms.

This is for rent alone. Board is estimated to cost 10s. a week; the term "board" including food, baths, linen, lights, and general service. The items are thus analysed:—

| | s. | d. |
|---|---|---|
| Seven breakfasts at 3½d. ... ... ... ... ... | 2 | 0½ |
| Six dinners at 8d. and one (Sunday) at 10d., with tea or coffee to follow ... ... ... ... ... | 4 | 10 |
| Extra meal (supper) on Sunday... ... ... ... | 0 | 6 |
| | 7 | 4½ |
| Leaving for use of baths, linen, lights, and general service ... ... ... ... ... ... ... | 2 | 7½ |
| | 10 | 0 |

This leaves to the resident to provide for herself luncheon, afternoon tea, and the like, which are very rarely taken at home. If wanted there, it is not difficult to make use of a spirit-lamp, and provide and wash a cup and plate. But, if the demand were sufficient, it might be possible to provide these in the house at a small extra charge.

The estimated cost is: For the buildings, £24,000; and for furniture, £2,400. The expenses of management are thus estimated:—

| | |
|---|---|
| Rates and taxes ... ... ... ... ... ... | £700 |
| Manager's salary and living... ... ... ... ... | 200 |
| Housekeeper's salary and living... ... ... ... | 150 |
| Cook and five kitchenmaids' salary and living ... | 250 |
| Sixteen house-parlourmaids' salary and living... ... | 768 |
| Kitchen porters, night porter, hall-boys ... ... | 600 |
| Passenger and service lifts ... ... ... ... | 170 |
| Depreciation, repairs, and insurance ... ... ... | 500 |
| Laundry (about) ... ... ... ... ... ... | 400 |
| | £3,738 |

To meet this the following receipts might be expected:—

| | |
|---|---|
| Rent of 70 double bedrooms at 8s. per week ... | ...£1,456 |
| " 50 " 7s. 6d. " ... | ... 1,021 |
| " 280 " 5s. " ... | ... 3,640 |
| | £6,117 |

This shows a profit of £2,379. Deduct from this £400 for ground rent, and reckon depreciation of furniture at 10 per cent. of the cost, equal to £240 per annum. This would leave a 5 per cent. profit for the shareholders, amounting to £1,320, and there would still remain £419 to be carried forward. It is probable that there would also be some profit on the catering department.

This is the scheme. It certainly offers more comfort for the money than the educated working woman for whom it is intended can now command. So far as one can judge from the success of other similar, but inferior schemes, it has a fair chance of paying. The remaining question is, Are there to be found people who are willing to risk the £25,000 needed to start the building?

